# Estimation of proteinuria as a predictor of complications of pre-eclampsia: a systematic review

**DOI:** 10.1186/1741-7015-7-10

**Published:** 2009-03-24

**Authors:** Shakila Thangaratinam, Arri Coomarasamy, Fidelma O'Mahony, Steve Sharp, Javier Zamora, Khalid S Khan, Khaled MK Ismail

**Affiliations:** 1 Academic Unit of Obstetrics and Gynaecology, Birmingham Women's Hospital, Birmingham, B15 2TG, UK; 2 Academic Unit of Obstetrics and Gynaecology, Keele University School of Medicine, University Hospital of North Staffordshire, Stoke-on-Trent, UK; 3 NLH Specialist Library for ENT and Audiology, John Radcliffe Hospital, Oxford, UK; 4 Clinical Biostatistics Unit, Hospital Ramón y Cajal, Department of Biomathematics, University Complutense of Madrid, Madrid, Spain

## Abstract

**Background:**

Proteinuria is one of the essential criteria for the clinical diagnosis of pre-eclampsia. Increasing levels of proteinuria is considered to be associated with adverse maternal and fetal outcomes. We aim to determine the accuracy with which the amount of proteinuria predicts maternal and fetal complications in women with pre-eclampsia by systematic quantitative review of test accuracy studies.

**Methods:**

We conducted electronic searches in MEDLINE (1951 to 2007), EMBASE (1980 to 2007), the Cochrane Library (2007) and the MEDION database to identify relevant articles and hand-search of selected specialist journals and reference lists of articles. There were no language restrictions for any of these searches. Two reviewers independently selected those articles in which the accuracy of proteinuria estimate was evaluated to predict maternal and fetal complications of pre-eclampsia. Data were extracted on study characteristics, quality and accuracy to construct 2 × 2 tables with maternal and fetal complications as reference standards.

**Results:**

Sixteen primary articles with a total of 6749 women met the selection criteria with levels of proteinuria estimated by urine dipstick, 24-hour urine proteinuria or urine protein:creatinine ratio as a predictor of complications of pre-eclampsia. All 10 studies predicting maternal outcomes showed that proteinuria is a poor predictor of maternal complications in women with pre-eclampsia. Seventeen studies used laboratory analysis and eight studies bedside analysis to assess the accuracy of proteinuria in predicting fetal and neonatal complications. Summary likelihood ratios of positive and negative tests for the threshold level of 5 g/24 h were 2.0 (95% CI 1.5, 2.7) and 0.53 (95% CI 0.27, 1) for stillbirths, 1.5 (95% CI 0.94, 2.4) and 0.73 (95% CI 0.39, 1.4) for neonatal deaths and 1.5 (95% 1, 2) and 0.78 (95% 0.64, 0.95) for Neonatal Intensive Care Unit admission.

**Conclusion:**

Measure of proteinuria is a poor predictor of either maternal or fetal complications in women with pre-eclampsia.

## Background

Pre-eclampsia is associated with increased maternal and fetal mortality and morbidity. Proteinuria is one of the essential criteria for the clinical definition of pre-eclampsia. It is part of the fundamental investigations performed by healthcare professionals in primary and secondary care to monitor disease severity and predict complications in women with pre-eclampsia. Urinalysis by visual reagent strip tests is widely performed in antenatal clinics and in the community by various health professionals. Total protein estimation in a 24-hour urine sample is also frequently used to assess the severity of pre-eclampsia in patients admitted to the hospital. More recently, spot urine protein:creatinine ratio has been used to provide an accurate quantification of 24-hour proteinuria [1]. Estimation of the accuracy of the predictive value of proteinuria by any of the above methods in predicting maternal and fetal complications will aid in clinical management by identifying the highest risk women who may need aggressive management, and the lower risk women in whom unnecessary interventions may be avoided.

Proteinuria occurs due to renal glomerular endotheliosis, a manifestation of widespread endothelial damage in pre-eclampsia [2,3]. The association between proteinuria and adverse fetal outcomes was first highlighted by Page and Christianson [4]. Since then, increased excretion of protein in women with pre-eclampsia has been generally associated with adverse maternal and fetal outcomes [5-7]. However, the primary diagnostic studies that evaluate the association between increase in the levels of proteinuria and maternal and neonatal outcomes have not generally been conducted with sufficiently large sample size to provide precise accuracy estimates. Moreover, they vary widely in their definition of pre-eclampsia, maternal and fetal outcomes, the method used for measurement and optimal cut-off levels of proteinuria. There are no systematic reviews exploring the accuracy of proteinuria to predict complications of pre-eclampsia. We therefore conducted a comprehensive systematic review to obtain precise estimates of likelihood ratios of adverse maternal and fetal complications for various cut-off levels of proteinuria in women with pre-eclampsia.

## Methods

### Data Sources

The review was carried out with a prospective protocol using widely recommended methods [8-12]. We searched MEDLINE (1951 to 2007), EMBASE (1980 to 2007), Cochrane Library (2007) and MEDION (a database of diagnostic test reviews set up by Dutch and Belgian researchers) for relevant citations. A search term combination was constructed after exhaustive planning and piloting of possible search concepts capturing the relevant population, tests and outcomes. Our search terms are shown in Additional file 1. An initial search in Medline yielded 11,711 citations. The search strategy was adapted for searching in Embase to obtain a total of 19,500 citations. From this citation set, studies were selected for inclusion in the review in a two-stage process if they studied the accuracy of proteinuria in the prediction of maternal and fetal complications in women with pre-eclampsia. The reference lists of all known primary and review articles were examined to identify cited articles not captured by electronic searches. Language restrictions were not applied. A comprehensive database of relevant articles was constructed.

### Study Selection

Studies which evaluated the accuracy of proteinuria in women with pre-eclampsia for the prediction of maternal or fetal complications were selected in a two-stage process. We included studies that pre-specified the patients to have pre-eclampsia, used bedside (urine dipstick) or laboratory methods (24-hour protein estimation, urine protein:creatinine ratio) to measure levels of proteinuria and assessed maternal or fetal clinical complications as outcome. First, the electronic searches were scrutinised and full manuscripts of all citations that were likely to meet the predefined selection criteria were obtained. Second, final inclusion or exclusion decisions were made by the reviewers (ST and FOM) after examination of these manuscripts. Studies which met the predefined and explicit criteria regarding population, tests, outcomes and study design (Additional file 2) were selected for inclusion in the review. When disagreements occurred, they were resolved by consensus (ST, KI and FOM). In cases of duplicate publication, the most recent and complete versions were selected. There were no language restrictions.

Information was extracted from each selected article on study characteristics, quality and accuracy results. Accuracy data were used to construct 2 × 2 tables of proteinuria result (test positive if proteinuria levels were above the threshold defined in the primary study, and test negative if these were below the threshold) and maternal and fetal outcomes.

### Methodological quality assessment

All manuscripts meeting the selection criteria were assessed for their methodological quality. Quality was defined as the confidence that the study design, conduct and analysis minimised bias in the estimation of test accuracy. Based on existing checklists [13,14], quality assessment involved scrutinizing study design and relevant features of the population, test and outcomes of the study. A study was considered to be of good quality if it used a prospective design, consecutive enrolment, full verification of the test result with reference standard, and had adequate test description. We excluded studies with case-control design as these are known to result in substantial bias [13].

### Data synthesis

Likelihood ratios (LRs) for positive and negative test results were calculated for each study, separately for each test threshold. Summary LRs were then computed where appropriate for positive and negative test results for each individual test threshold and for each outcome of interest. This information is clinically more relevant than traditional summaries of accuracy such as sensitivity and specificity, as LRs allow the estimation of post-test probabilities of various complications for women at different risk levels [15]. The LR indicates by how much a given test result raises or lowers the probability of having the disease. The higher the LR of an abnormal test, the greater is the value of the test. Conversely, the lower the LR of a normal test, the greater is the value of the test. An LR of >10 or <0.1 is regarded as 'very useful' test accuracy, whilst an LR of 5 to 10 or 0.1 to 0.2 is regarded as 'moderately useful', and an LR of 2 to 5 or 0.2 to 0.5 is regarded as 'somewhat useful'. An LR of 1 to 2 or 0.5 to 1 is only regarded as 'little useful' and an LR of 1 as 'useless'. Although, this categorization is useful for interpretation of LRs, it should be noted that the value of a test may vary depending on the pre-test probability of the condition, and the consequences of treatment.

Heterogeneity of diagnostic odds ratio (dOR) was assessed graphically using forest plot [16] (not shown) and statistically using chi-squared test [17] to aid in decisions on how to proceed with quantitative synthesis [18]. As, for some tests and outcomes, there was either graphical or statistically significant heterogeneity, we used random effects model meta-analysis [17]. Where a quantitative approach was not appropriate due to significant clinical heterogeneity, we refrained from pooling and the results are described narratively and the LR of maternal and fetal complication estimated in each study is reported. All statistical analyses were performed using Meta Disc statistical package.

## Results

### Literature identification and study quality

**Figure 1:**
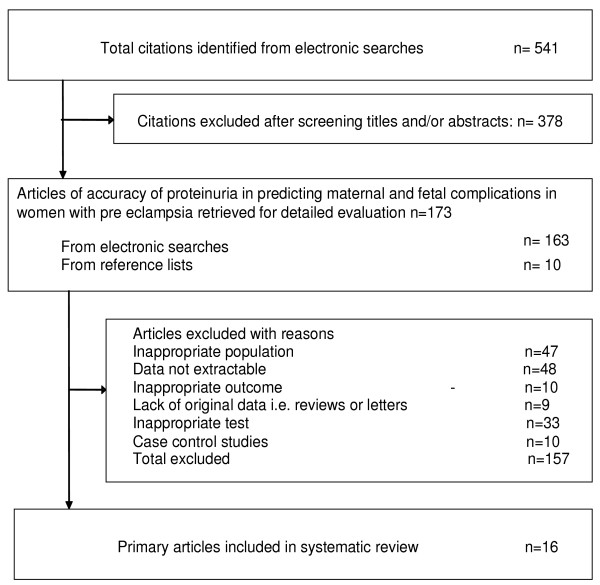
**Study selection process for systematic review of proteinuria to predict maternal and fetal complications**.

**Figure 2:**
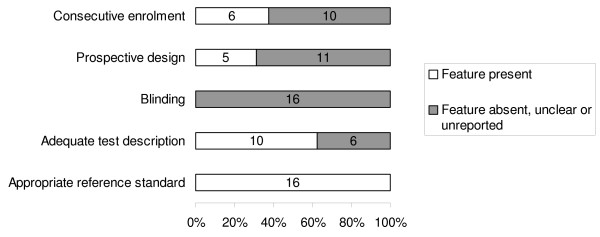
**Quality of the included primary studies in the systematic review of accuracy of proteinuria in predicting complications in women with pre-eclampsia**.

Figure 1 summarizes the process of literature identification and selection. There were 16 primary articles that met the selection criteria including a total of 6749 women (Figure 1) [1,19-34]. Eight articles reported estimation of proteinuria by laboratory method only [20,21,23,26,28,29,33,34], five by bedside dipstick urinalysis only [22,24,25,30,32], two by either of the above methods [27,31] and one by spot urine protein:creatinine ratio [1]. The salient features (population subgroups, test characteristics and reference standards) of each individual study can be obtained from the authors. The definition of pre-eclampsia differed widely between the studies. The test threshold in individual studies for laboratory estimation varied from 0.3 g/24 h to 10 g/24 h, or was reported as an increase in proteinuria by 2 g/24 h between two measurements. The cut-off levels for bedside urinalysis using visual reagents ranged from 1+ to 4+ of proteinuria. One study evaluated the accuracy of spot urine protein: creatinine ratio for threshold levels of 500 mg/mmol and 900 mg/mmol in the prediction of maternal and fetal complications [1]. The methodological quality of the included studies is given in Figure 2.

### Proteinuria to predict maternal outcomes

**Figure 3:**
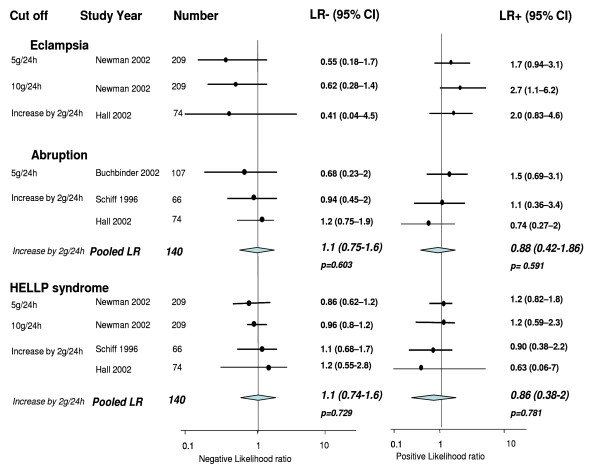
**Likelihood ratios for maternal outcomes in women with pre-eclampsia for various threshold levels of proteinuria**.

Three test accuracy studies evaluated the accuracy of proteinuria in predicting eclampsia for cut off levels of 5 g/24 h, 10 g/24 h and an increase by 2 g in 24 hours [23,26]. The LR of positive test ranged from 1.7 (95% CI 0.94, 3.1) to 2.7 (95% CI 1.1, 6.2) respectively. The negative LR ranged from 0.41 (95% CI 0.04, 4.5) to 0.62 (95% CI 0.28, 1.4) (Figure 3).

Two studies estimated the accuracy of proteinuria in predicting placental abruption using a cut-off of increase in level more than 2 g/24 h [23,29]. The pooled positive and negative LRs for the above cut-off were 0.88 (95% CI 0.42, 1.86) and 1.1 (95% CI 0.75, 1.6) respectively (Figure 3).

HELLP syndrome prediction was evaluated in four test accuracy studies [23,26,29]. The pooled estimate of LRs for positive and negative test for a cut-off of increase in levels more than 2 g in 24 hours were 0.86 (95% CI 0.38, 2) and 1.1 (95% CI 0.74, 1.6) respectively (Figure 3).

### Proteinuria to predict fetal outcomes

#### Fetal, neonatal and perinatal mortality

**Figure 4:**
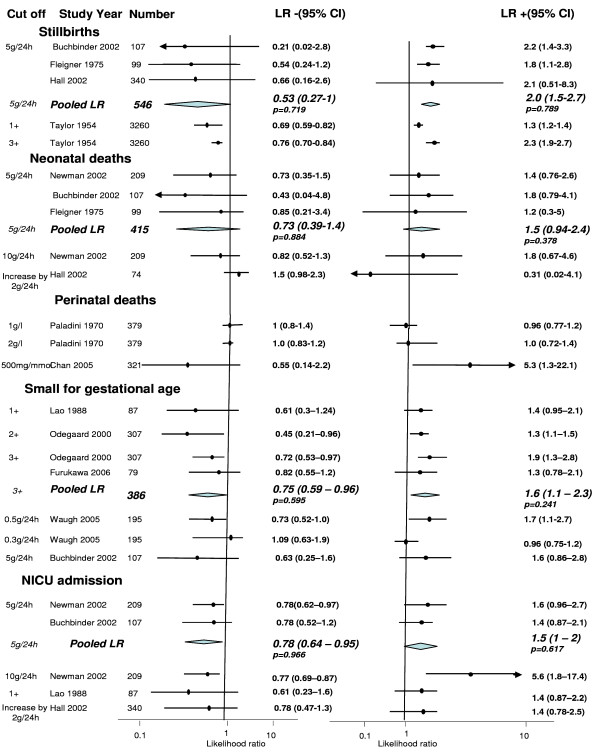
**Likelihood ratios for fetal outcomes in women with pre-eclampsia for various threshold levels of proteinuria**.

Thirteen studies reported prediction of fetal, neonatal and perinatal mortality using both laboratory and bedside testing for proteinuria [1,20,21,23,26,28-30,34]. The pooled LRs for positive and negative test for a cut-off of 5 g/24 h as reported in three studies [20,21,23] were 2 (95% CI 1.5, 2.7) and 0.53 (95% CI 0.27, 1) respectively (Figure 4). The largest study, involving 3260 patients, that estimated the prediction of stillbirths in pre-eclampsia was conducted by Taylor et al using urine dipstick method [35].

Neonatal deaths were evaluated in five studies for cut-off levels of 5 g/24 h (*n *= 3) [20,21,26], 10 g/24 h (*n *= 1) [26] and increase by 2 g in 24 h (*n *= 1) [23]. The pooled estimates of LR+ and LR- for proteinuria threshold of 5 g/24 h were 1.5 (95% CI 0.94, 2.4) and 0.73 (95% 0.39, 1.4) respectively.

The threshold levels of proteinuria to predict perinatal deaths were 1 g/l, 2 g/l and 500 mg/mmol. The positive LR was 5.3 (95% CI 1.3, 22.1) and the negative LR was 0.55 (95% CI 0.14, 2.2) for cut-off levels of 500 mg/mmol [1].

#### Small for gestational age

Four studies assessed the accuracy of bedside urinalysis for cut-offs of 1+, 2+ and 3+ of proteinuria in urine dipstick [22,24,27]. The pooled positive and negative LRs for 3+ of proteinuria were 1.6 (95% CI 1.1, 2.3) and 0.75 (95% CI 0.59, 0.96) respectively. The likelihood ratio of laboratory estimates of proteinuria levels of 0.3 g/24 h and 0.5 g/24 h were 0.96 (95% CI 0.75, 1.2) and 1.7 (95% CI 1.1, 2.7) for positive test and 1.09 (95% 0.63, 1.9) and 0.73 (95% CI 0.52, 1) for negative test respectively, using the benzethonium chloride assay (BCA) [33]. For tests performed on the same patients using the Bradford assay Waugh et al report positive likelihood ratios of 1.71 (95% CI 1.0, 2.9) and 2.79 (95% CI 1.4, 5.5) for cut-off levels of 0.3 g/24 h and 0.5 g/24 h respectively [33].

#### Neonatal Intensive Care Unit (NICU) admission

NICU admission was assessed as an outcome in five studies [20,23,24,26]. The pooled positive and negative LRs for cut-off level of 5 g/24 h were 1.5 (95% CI 1, 2) and 0.78 (955 CI 0.64, 0.95) and for levels of 10 g/24 h, the LRs were 5.6 (95% CI 1.8, 17.4) and 0.77 (95% CI 0.69, 0.87) respectively.

## Discussion

Proteinuria has usually been associated with increase in maternal and fetal mortality and morbidity [7]. Our review has shown that the magnitude of proteinuria in women with pre-eclampsia is a poor predictor of the major maternal and fetal complications.

For prediction of adverse fetal outcomes, the only statistically significant results were observed for positive test result with LR+ ranging from 1.3 to 2.3 for cut-off levels of 5 g/24 h, 1+ and 3+ proteinuria in the prediction of stillbirths [20,21,23,30]. Furthermore, we need to take into account that three of these five test accuracy studies were conducted more than 30 years ago [21,30]. The test was found to be a poor predictor of neonatal and perinatal deaths with no significant LRs for positive or negative test. The test performed poorly as evidenced by the increase in adverse events noticed in the test negative group compared with the test positive group, as noticed in some studies [23,28,33]. The overall low value of abnormal test and high value of normal test implies that the test is of 'very little' clinical value.

The validity of our review findings depends on the methodology of the systematic review and the quality of the individual studies included [13]. An extensive literature search was performed in relevant databases without any language restrictions to minimize the possibility of missing any studies. Methodological deficiencies such as verification bias, differential use of reference standards and case-control design did not apply to the studies in the review, ensuring inclusion of studies of acceptable quality.

The methodological problems facing reviews of this nature are daunting. A significant limitation of this review is the heterogeneity noticed between individual studies with regards to population, definition of pre-eclampsia, method of performing the test, test thresholds, frequency of testing, interval between the test and outcome, and reference standards. The lack of information regarding the temporal relation between test findings and outcomes observed and possibility of confounding by other risk factors contributing to maternal and fetal complications may influence the observed predictive value of proteinuria for maternal and fetal complications. The wide confidence intervals observed for the various outcomes are a reflection of the statistical uncertainty around the results due to the small sample size in many studies. Meta-analysis of studies using individual patient data may conquer many of the difficulties identified.

## Conclusion

This systematic review has shown that estimation of levels of proteinuria in women with is not a clinically useful test to predict fetal or maternal complications. The results of this review calls into question the commonly used practice of making clinical decisions in women with pre-eclampsia based on the severity of proteinuria. It has highlighted the need for large, well-designed prospective studies on this important question with the hope to expand future research.

## Competing interests

The authors declare that they have no competing interests.

## Authors' contributions

KSK conceived the idea of the review and developed the protocol with KMKI, FOM, AC and ST. SS searched the electronic databases to identify the studies. JZ and ST conducted statistical analysis. All authors contributed to the writing of manuscript.

## Pre-publication history

The pre-publication history for this paper can be accessed here:



## Supplementary Material

Additional file 1 **Search term combinations for identification of studies predicting complications of pre eclampsia.** Click here for file

Additional file 2 **Study characteristics of the trials included in the systematic review of accuracy of proteinuria in predicting complications in women with pre-eclampsia.** Click here for file
