# Supplementary material for: Estimation of proteinuria as a predictor of complications of pre-eclampsia: a systematic review
Source: BMC Med. 2009 Mar 24;7:10. doi: 10.1186/1741-7015-7-10 (PMC2670320; doi:10.1186/1741-7015-7-10)
Supplement: Additional file 1 — Search term combinations for identification of studies predicting complications of pre eclampsia. [file 1741-7015-7-10-S1.doc]

Table 1: Search term combinations for identification of studies predicting complications of pre eclampsia

**Fig 1. Flow diagram of study selection process in Medline**

| Population | Test | | | Outcome | Final |
| --- | --- | --- | --- | --- | --- |
|  | History | Examination | Investigation |  | Refinement |
| 1. pre adj eclampsia  2. preeclampsia  3. hypertens$  4. pregnan$  5. pre-eclampsia#.DE.  6. hypertension#.DE.  7. pregnancy#.DE***.***  8. 3 or 6(hypertension)  9. 4 OR 7(pregnancy)  10. 8 and 9(pregnancy and hypertension) | 12. history  13. parity  14. multiparity or nulliparity  15. matern$ near age  16. (previous or prior) near eclampsia  17. (previous or prior) near preeclampsia  18. (previous or prior) near pre adj eclampsia  19. multiple near pregnan$*  20. twin$ or triplet$ or quadruplet$  21. symptom$  22. headache  23. epigastric near pain  24. naus$ or vomit$  25. race  26. diabet$  27. stress  28. lupus  29. thrombophilia  30. medical-history-taking#.DE.  31. maternal-age#.DE.  32. pregnancy-multiple#.DE.  33. headache#.DE.  34. signs-and-symptoms-digestive#.DE.  35. vision-disorders#.DE.  36. weight gain#.DE.  37. population-groups#.DE.  38. diabetes-mellitus#.DE.  39. stress-psychological#.DE.  40. autoimmune-diseases#.DE.  41. thrombophilia#.DE. | 43. blood adj pressure  44. oedema or edema  45. tendon$ near reflex$  46. hyperreflexia  47. clonus  48. papilledema or papilloedema  49. retina$ near change$  50. oliguria  51. symphys$ near fundal  52. symphys$ near height  53. cardiotocogra$  54. oxygen near saturat$  55. blood-pressure-determination#.DE.  56. edema#.DE.  57. reflex-abnormal#.DE.  58. retinal-diseases#.DE.  59.oliguria#.DE.  60. cardiotocography#.DE.  61.oximetry#.DE. | 63. serum near uric adj acid  64. urin near analys$  65. urin$  66. maternal near (feto adj protein$ or fetoprotein$ or alphafetoprotein$)  67. urin$ near calcium  68. hypoalbuminemia or hypoalbuminaemia  69. microalbuminuria  70. fibronectin$  71. proteinuria  72. renal adj function near test$  73. liver adj function near test$  74. liquor near volume  75. biophysical near profile  76. ultraso$  77. antithrombin$  78. platelet adj count  79. anti adj thrombin$  80. fibrinogen  81. antiphospholipid$  82. haemoglobin  83. uric-acid-QN.DE  84. alpha-fetoproteins#.DE  85. calcium-ur.DE  86. hypoalbuminemia#.DE.  87. fibronectins.DE.  88. proteinuria#.DE.  89. kidney-function-tests#.DE.  90. liver-function-tests#.DE.  91. ultrasonography#.DE.  92. haematologic-tests#.DE.  93. antithrombin-III.DE.  94. fibrinogen#.DE.  95. antibodies-antiphospholipid#.DE.  96. diagnostic-imaging#.DE. | 99. complicat$  100. (renal or kidney$) near (disease$ or complicat$)  101. (hepatic or liver$) near (disease$ or complicat$)  102. death or mortality  103. morbidity  104. eclampsia  105. (pulmonary or lung) near (complicat$ or disease$)  106. thromboembolism  107. pulmonary near(oedema or edema)  108. ventilat$  109. stroke  110. uter$5 near haemorrhage  111. abruption  112. (heart or cardiac) near arrest$  113. (psychiatric or mental) near (illness$ or complication$1 or disorder)  114. hospitali$  115. hypox$ near isch$  116. (development$ or learning) near (disorder$ or difficult$)  117. pregnancy-complications#.DE.  118. kidney-diseases#.DE.  119. renal-dialysis#  120. liver-diseases#.DE.  121. death#  122. eclampsia#  123. pulmonary-embolism.DE.  124. respiration-artificial#  125. cerebrovascular-disorders#.DE.  126. brain-edema.DE.  127. intracranial-hypertension#.DE.  128. uterine-haemorrhage.DE.  129. abruption-placentae#.DE.  130. heart-diseases#  131. mood-disorders#.DE.  132. hospitalization#  133. infant-newborn-diseases#  134. respiratory-distress-syndrome-newborn.DE.  135. mental-disorders-diagnosed-in-childhood#.DE. | 1. 11 and 98 and 136   (Captures *Population* and *Test* and *Outcome***)**  138. animal=yes  139. human=yes  140. 138 not 139  141. 137 not 140  142. PT= comment or PT= letter |
| **11. 1 OR 2 OR 5 OR 10**  **(Captures *Population*)** | **42. OR/12-41**  **(Captures history)** | **62. or/43-61**  **(Captures examination)** | **97. or/63-96**  **(Captures investigation)** | **136. or/99-135**  **(Captures *Outcome*)** | **143. 141 not 142**  ***Final citation set (animal only studies, comments and letters excluded)*** |
|  | **98. 42 or 62 or 97**  **(Captures *Test*)** | | |  |  |

***Key to commands and codes used in Dialog interface:***

*Adj = words adjacent;; near = words within five words of each other in any order;.DE.=descriptor (MeSH heading);# =Exploded MeSH heading*

*$ =Truncated to allow for variant word endings; QN =Quick analysis pre-exploded subheading (including analysis, blood, urine, cerebrospinal fluid, isolation and purification PT = Publication Type*
