# Supplementary material for: Estimation of proteinuria as a predictor of complications of pre-eclampsia: a systematic review
Source: BMC Med. 2009 Mar 24;7:10. doi: 10.1186/1741-7015-7-10 (PMC2670320; doi:10.1186/1741-7015-7-10)
Supplement: Additional file 2 — Study characteristics of the trials included in the systematic review of accuracy of proteinuria in predicting complications in women with pre-eclampsia. [file 1741-7015-7-10-S2.doc]

| **Study**  **Table 2. Study characteristics of the trials included in the systematic review of accuracy of proteinuria in predicting complications in women with pre-eclampsia** | | **Population** | | | | | **Test** | | **Outcome** |
| --- | --- | --- | --- | --- | --- | --- | --- | --- | --- |
| **Study(Year)**  **Language** | **Quality** | **Number of patients** | **Inclusion criteria** | **Exclusion criteria** | **Any intervention** | **Gestation of testing** | **Frequency of testing** | **Cut off level** |
| Newman (2002)  English | Cross-sectional  Not blind  Consecutive  Retrospective  Test not described | 209 | Pre-eclampsia (ACOG definition) and chronic hypertension without proteinuria early in pregnancy | Multiple pregnancy, renal disease | Delivery if BP>=160/110, HELLP syndrome or fetal distress | Not Known | Within 48 hours of admission  Freq?? | >=5g/24h  >=10g/24h | **Eclampsia**  **Severe HT**  **5’Apgar<7**  **HELLP syndrome**  **Neonatal death**  **NICU admission**  **Del<32w**  **RDS**  **IVH**  **NEC** |
| Buchbinder (2002) English | Cross-sectional  Not Blind  Prospective  Test not described  Arbitrary | 107 | Singletons, previous H/O pre-eclampsia, BP≥140/90 on 2 occasions 4 hours apart or 1 DBP>=110 mm Hg, proteinuria>=300mg/24h or 2 dipstick >=2+ (100mg/dl) 4h apart with no evidence of UTI | Multiple pregnancy, DM, chronic HT, baseline proteinuria | Low dose aspirin | Not Known | Frequency not known | 5g/24h | **Preterm delivery**  (delivery <37 weeks)  **Fetal death**  **Neonatal death**  **Abruptio placentae (APH+uterine tenderness+placental examination)**  **NICU admission**  **IVH**  **RDS**  **Small for gestational age (Brenner table)** |
| RA Odegaard  (2000)  English | Cross-sectional  Not blind  Retrospective  Enrolment not described  Test described | 307 | Increase in DBP of 25mm Hg to at least 90 mm Hg and proteinuria after 20w | Multiple pregnancy, Unknown GA | Not available | Not Known | NA | 2+  3+ (500 mg/24h) | **Small for gestational age**  ( 2 SD below EFW or >24%lower than expected BWt or 840g reduction in B Wt for term infant) |
| Schiff  (1996)  English | Cross-sectional  Not blind  Retrospective  Enrolment not described  Test not described | 66 | Severe pre-eclampsia (BP>140/90 and proteinuria >=300mg/24h and hyperuricemia ua >5mg/dl and one of following- SBP>=160 or DBP>=110, proteinuria >=5g/24h, AST >72 U/L) between 26 and 32 w | Chronic HT | Delivery if thrombocytopenia (<100,000/ ml), uncontrolled HT or persistent symptoms | Between 26 and 32 weeks | Two tests 4 or more days apart after admission | Increase in 24h prot by >=2g | **HELLP**  **CS for fetal distress**  **Abruption**  **Eclampsia**  **Stillbirth**  **5’ Apgar <=6** |
| Von Dadelzen  (2004)  English | Cross-sectional  Not blind  Retrospective  Consecutive enrolment  Test described | 594 | Women admitted with at least 2 of the following  Hypertension (Systolic BP >=140 and/or DBP>=90 MM Hg 2 readings 4 hours apart) after 20 weeks  Proteinuria (>=0.3g/day or >=2+ dipstick) after 20 weeks  HELLP syndrome  Isolated eclamptic seizure without preceding hypertension or proteinuria | Women in spontaneous labour  Maternal outcome achieved before fulfilling eligibility criteria | Not available | Not available | Last observation carried forward | 1+, 2+, 3+ ,4+ 5+ | **Adverse maternal outcome**  **(death or complication involving hepatic or central nervous system or renal or respiratory or hematological systems )** |
| Hall (2002)  English | Cross-sectional  Not blind  Prospective  Test described  Not consecutive enrolment | 340 | ISSHP definition of PE  Singleton with early onset severe PE( >=24w, <34w) with heavy proteinuria (>=5g/24h) | N/A | Steroids 27-33w  MgSO4 eclampsia or imminent sym IP  Anti- HT to maintain BP at 160/110 mm Hg  Del at 34w if major mat or fetal complications | 24-34w | Twice weekly | Increase by 2g/24h in two samples | **Eclampsia**  **Abruption**  **HELLP**  **CS**  **Pulmonary oedema**  **ITU admission**  **Ascites**  **Fetal death**  **Low apgar**  **NNIC admission** |
| Taylor(1954)  English | Cross-sectional  Not blind  Retrospective  Test not described  Consecutive enrolment | 3258 | Toxemia with previous normal BP observations  Toxemia > 28w previous observations not known | Vascular or renal disease | Not available | Not available | Not available | 1+, 3+ | **Intra uterine death** |
| Thurnau  (1982)  English | Cross-sectional  Not blind Direction of data collection not available  Not consecutive enrolment  Test described | 83 | Pre eclampsia ACOG definition  Patients>24w | Not available | Not available | Not available | Not available | 1+, 2+, 3+, 5g | **Severe PE (ACOG definition)** |
| Martin (1999)  English | Cross-sectional  Not blind  Retrospective  Not consecutive enrolment  Test described | 568 | Patients severe pre-eclampsia (ACOG definition) | Eclampsia | Not available | On admission | Not available | 2+, 3+ | **Significant maternal morbidity**  Renal, hepatic and/or gastrointestinal |
| Paladini 1970  Italian | Cross-sectional  Not blind  Retrospective  Not consecutive enrolment  Test not described | 379 | Pre eclampsia defined as gestosis with systolic BP>140 and proteinuria>0.5g/l and oedema in 3^rd^ trimester | Only 1 or 2 symptoms of gestosis | Not available | 3^rd^ trimester | Not available | 1g/l  2g/l | **Perinatal death** |
| Fleigner 1975 | Cross-sectional  Not blind  Prospective  Enrolment not described  Test not described | 99 | Patients with moderate or severe pre-eclampsia between 30 and 37 weeks | Multiple pregnancy  Diabetes  Stillbirth on admission  Erythroblastosis | Anti-hypertensives  Magnesium sulphate  Anticonvulsants  Parenteral sedation  Diuretics | 30-37 weeks | Not available | 1-5g/l | **Stillbirth**  **Neonatal death**  **Perinatal death** |
| Lao 1988 | Cross-sectional  Not blind  Prospective  Consecutive enrolment  Test described | 87 | Nulliparous patients with pre-eclampsia (ACOG definition of patients with BP >=140/90mm Hg) | Multiple pregnancy  Diabetes  Untreated urinary infection  Renal disease  Chronic hypertension  Systemic Lupus Erythematosis (SLE) | Anti-hypertensives  if DBP>=100 mm Hg on 2 or more occasions | Not available | 4 times a day | 1+ | **Caesarean section**  **Small for gestational age**  **Neonatal Intensive Care Unit admission**  **5’ Apgar<4** |
| Weenik 1983 | Cross-sectional  Not blind  Direction of data collection not available  Enrolment not described  Test described | 57 | Pre-eclamptics with DBP>=100 mm Hg or 20 mmHg above non pregnant levels | Not available | Not available | Not available | Not available | 0.5, 1,2,3,4,5 g/24h | **Perinatal death or Neonatal Intensive Care Unit admission** |
| Chan 2005 | Cross-sectional  Not blind  Retrospective  Consecutive enrolment  Test described | 321 | Pre-eclampsia (ISSHP definition)  Hypertension (Systolic BP >=140 and/or DBP>=90 mm Hg) and Proteinuria (>=0.3g/24 hrs or a spot urine protein/creatinine ratio>=30 mg/mmol) after 20 weeks | Pre-eclampsia superimposed on pre-existing hypertension, unavailable spot urine results, booking BP>=140/90 mm Hg, postpartum diagnosis | Not available | Not Known | Not Known | Spot urine protein/ creatinine ratio  500 mg/mmol  900 mg/mmol | **Adverse maternal outcome**  **Adverse fetal outcome**  **Perinatal mortality** |
| Waugh 2005 | Cross-sectional  Not blind  Prospective  Consecutive enrolment  Test described | 195 | Sustained diastolic BP>=90mmHg, or a systolic BP of >=140 mmHg on 2 occasions or a single diastolic BP of >110 mmHg or systolic BP>160 mm Hg in women over 20 wks gestation | Less than 20 weeks gestation | Not available | Not Known | Not Known | 0.3g /24 h  0.5 g/24 h  (Benzethonium chloride assay) | **Small for gestational age** |
| Furukawa 2006 | Cross-sectional  Not blind  Retrospective  Not Consecutive enrolment  Test described | 79 |  |  |  |  |  | 3+ | **Small for gestational age** |
